# Supplementary material for: Single cell transcriptomic analysis of HPV16-infected epithelium identifies a keratinocyte subpopulation implicated in cancer
Source: Nat Commun. 2023 Apr 8;14:1975. doi: 10.1038/s41467-023-37377-0 (PMC10082832; doi:10.1038/s41467-023-37377-0)
Supplement: Supplementary file 1 — Supplementary Information [file 41467_2023_37377_MOESM1_ESM.pdf]

Supplementary Information

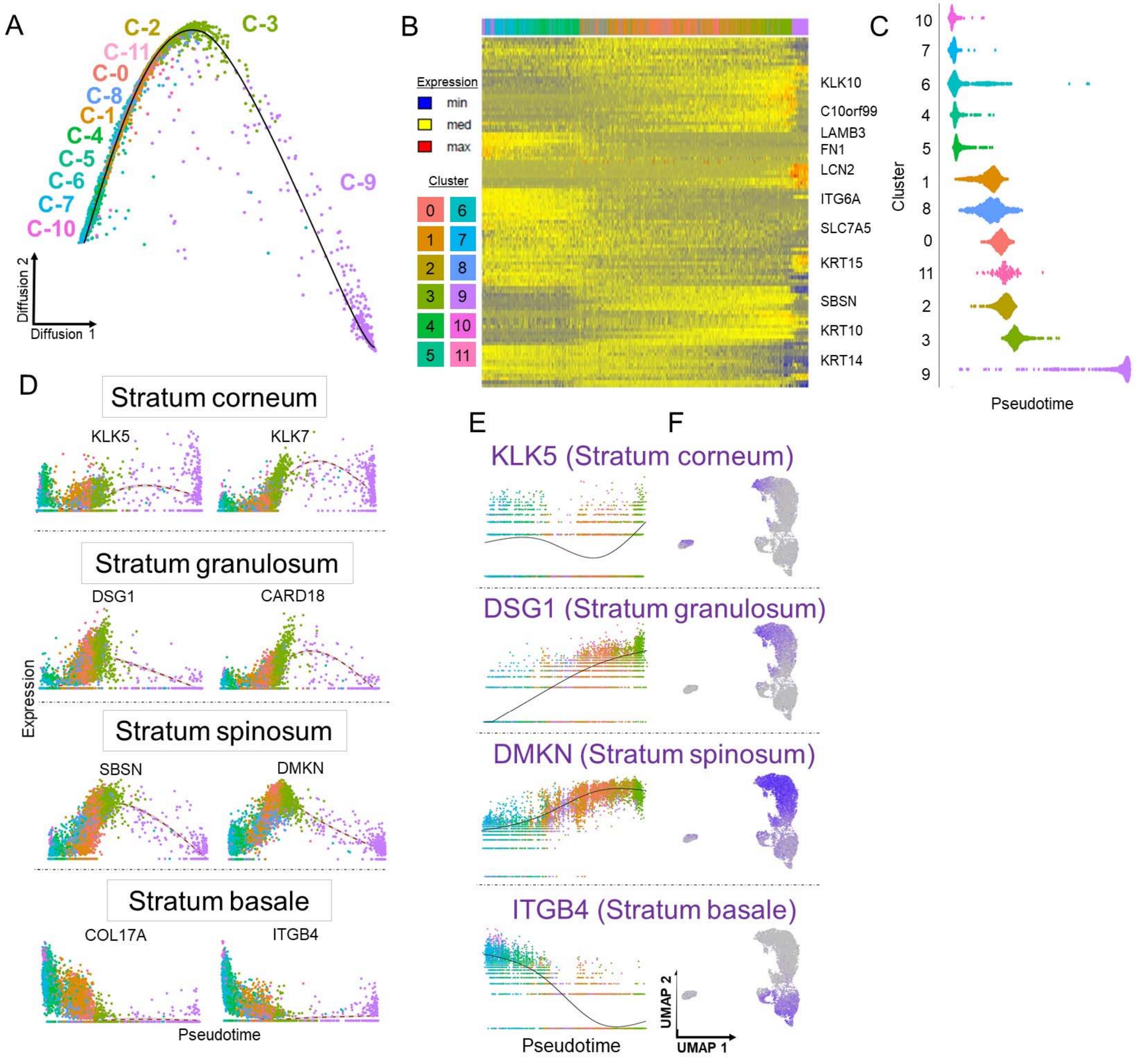

Supplementary Figure 1. Pseudotemporal trajectory analysis including C9 and additional plots to support Fig 3. a Pseudotemporal trajectory analysis, including the divergent C9 subpopulation, demonstrates that C9 does not fall in the linear trajectory (n=1 matched set). **b** Pseudotime heatmap with select highlighted genes reflects a progression from basal to differentiated cell types, with the exception of C9 whose transcriptome drastically diverges from conventional markers of the layers of the stratified squamous epithelium. **c** The pseudotime violin plot for analysis including C9 splits the majority of clusters into primarily early (basal) or late (differentiated) stages in the trajectory. **d** Gene expression over pseudotime of established markers of stratified epithelial layers confirm a progression from basal to differentiated populations with low expression in C9**.** From the pseudotime analysis excluding C9, additional markers of the layers of the stratified squamous epithelium are shown over pseudotime (**e**) and UMAPs (**f**) to supplement those shown in Fig. 3D and 3E.

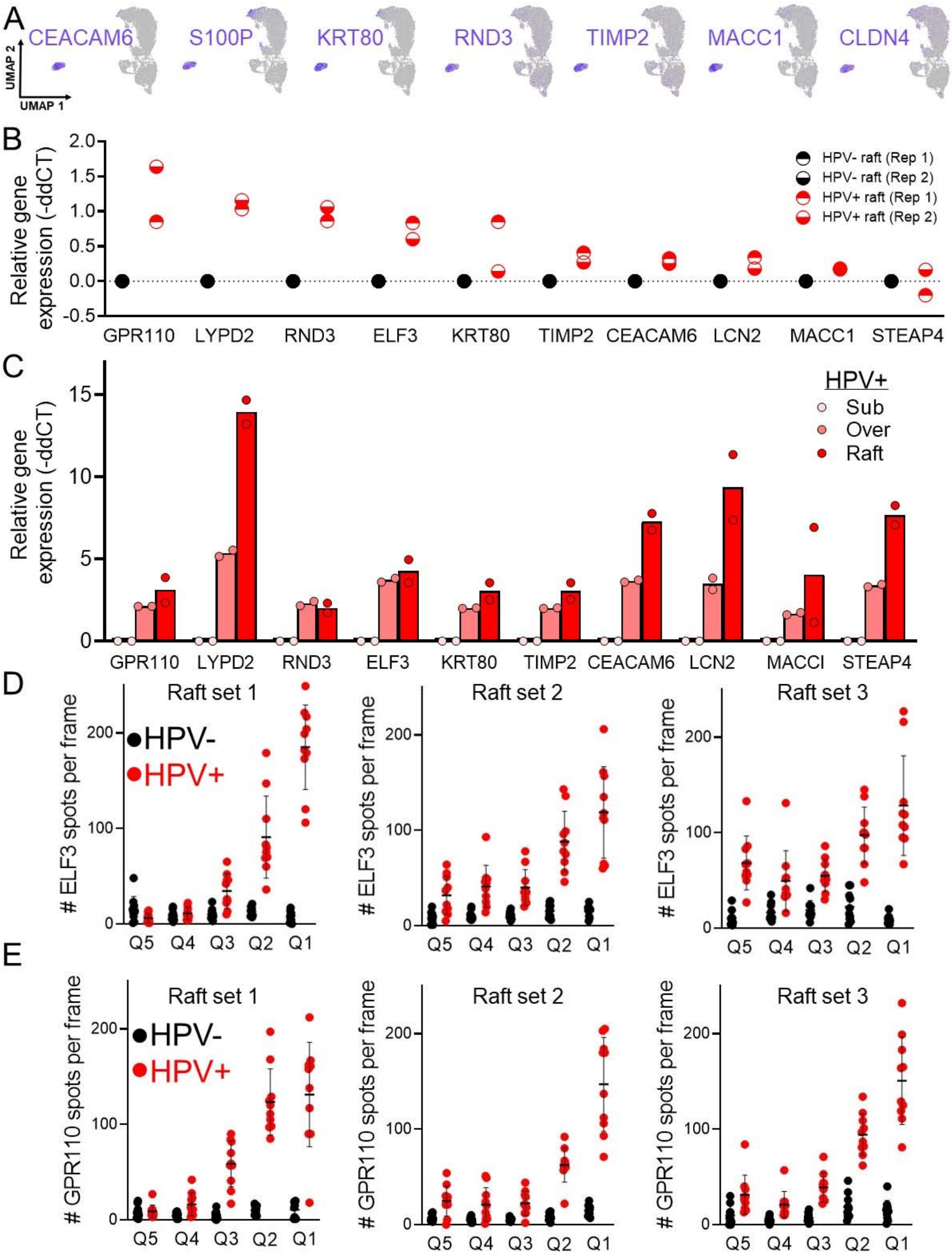

Supplementary Figure 2. Supportive Figure 5 data. a Feature plots of additional C9 biomarkers (n=1 matched set). **b** Higher expression of C9 biomarkers are observed overall in HPV+ vs HPV- rafts by RT-qPCR (n=2 rafts, with bars representing the mean). **c** C9 biomarkers are increased with differentiation, with C9 biomarkers increasing from subconfluency (undifferentiated) to overconfluency to 3D rafts in HPV16+ cells (n=2 rafts). Independent quantifications of ELF3 **(d)** and GPR110 **(e)** puncta by quintile per raft, where Q5 is the basal and Q1 is the uppermost raft layer, shows spatial localization of ELF3 expression in the suprabasal compartment (n=10 frames per raft). Data are represented as mean values +/- SD. Source data are provided in the Source Data file.

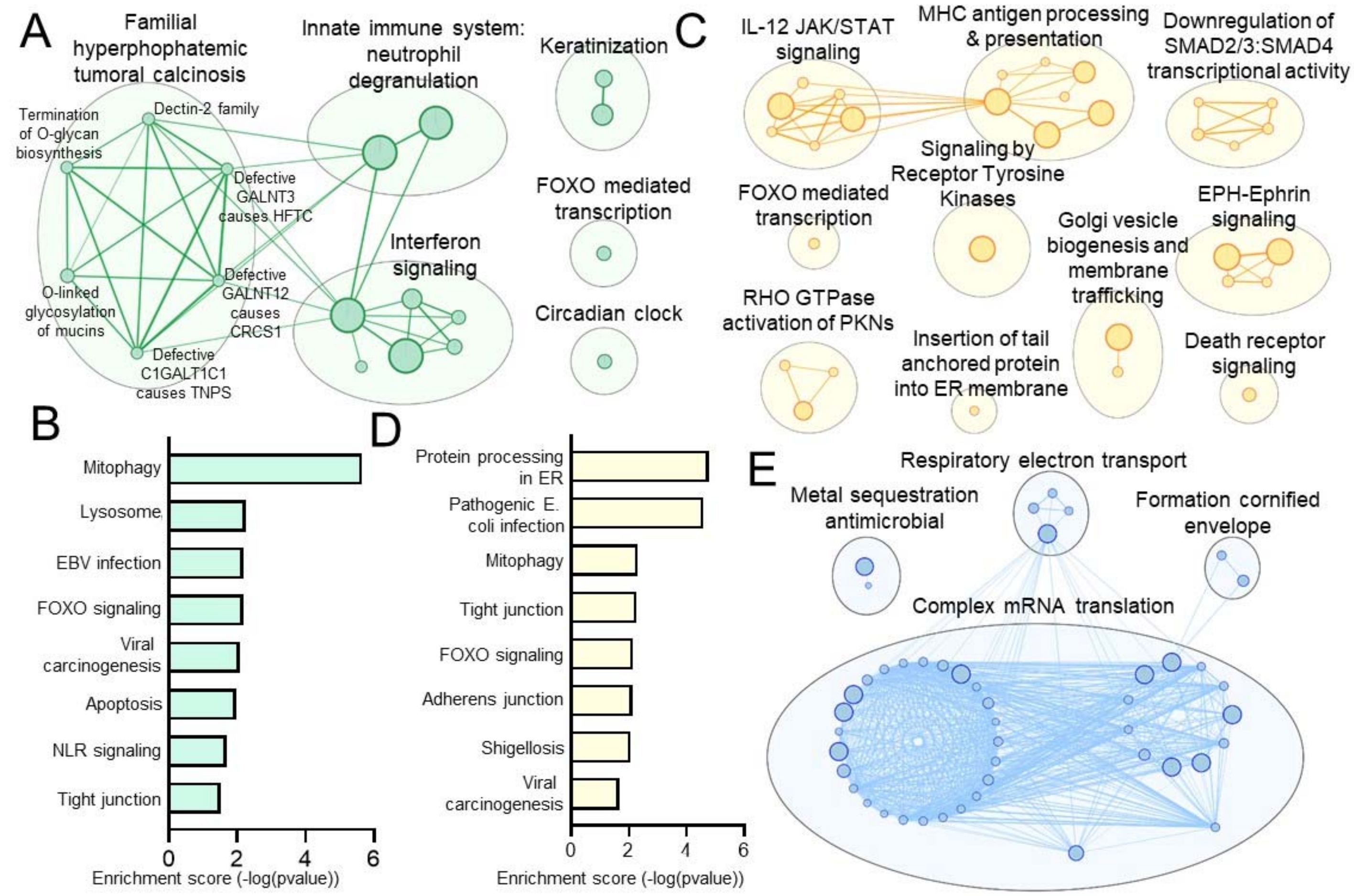

Supplementary Figure 3. Characterization of the C9 transcriptome. Gene ontologies were defined through the gProlifer web server, as detailed in Methods, which uses a tailor-made algorithm for statistical testing. **a** Cytoscape visualization of Reactome pathway analysis for the C9 transcriptome includes keratinization. **b** Summary of KEGG pathway hits for the C9 transcriptome include viral carcinogenesis and tight junction pathways. Differential expression of HPV+ vs HPV- rafts for C9 cells was then determined. **c** Cytoscape visualization of differential expression analysis for genes upregulated in HPV16+ versus HPV16- C9 cells includes immune signaling. **d** Upregulated KEGG pathways in HPV16+ versus HPV16- C9 cells include viral infection, junctions, and viral carcinogenesis. **e** Cytoscape visualization of differential expression analysis for genes downregulated in HPV16+ versus HPV16- C9 cells using Reactome pathway analysis. (**b,d**) Source data are provided in the Source Data file.

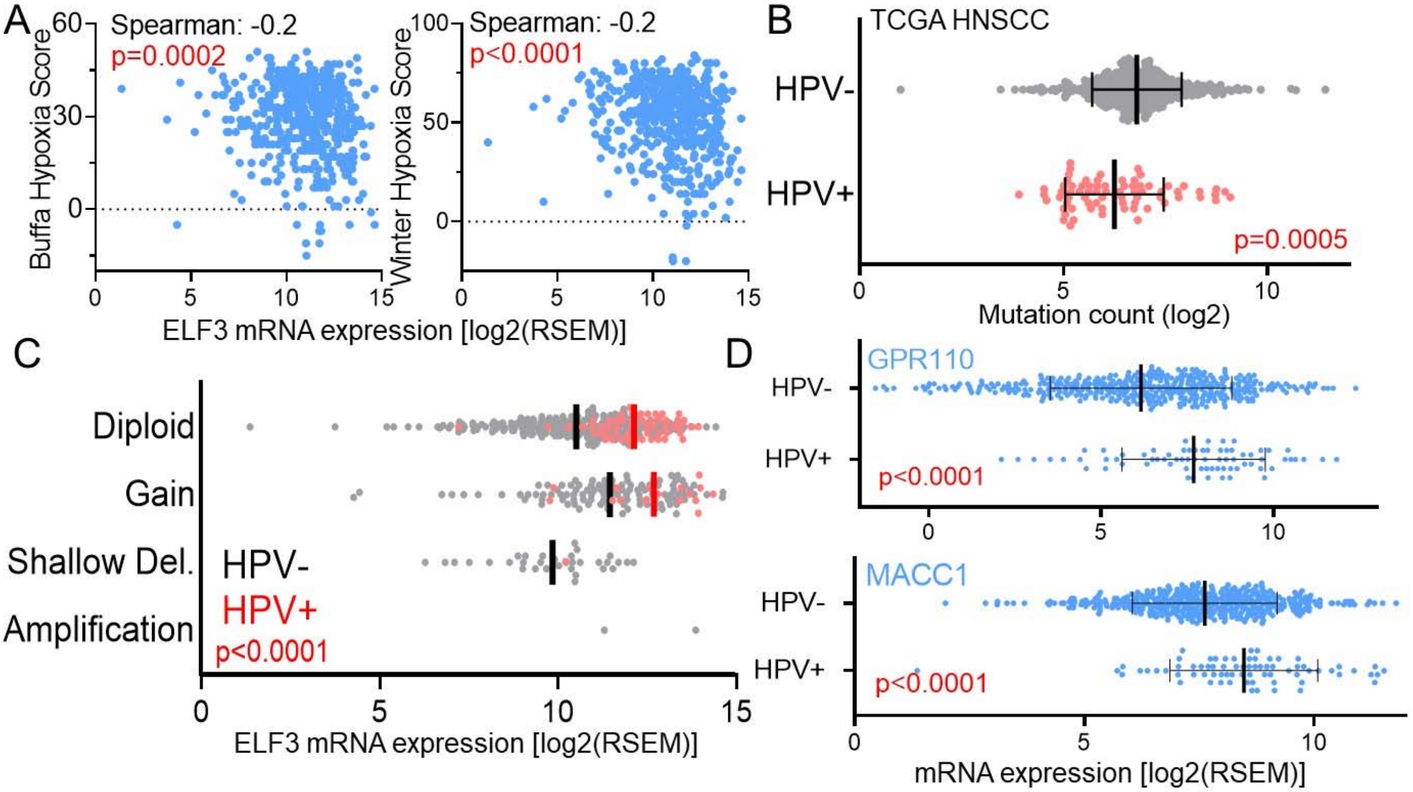

Supplementary Figure 4. Extended TCGA analyses of C9. a A negative correlation is observed between ELF3 mRNA expression and Buffa hypoxia score (r = -0.2, p < 0.0001, two-tailed Spearman r correlation) and Winter hypoxia score (r = -0.2, p < 0.001, Spearman rank correlation, n=515 tumors). (**b-d)** Analysis of 72 HPV+ and 415 HPV- tumors with available data in the TCGA. Data are represented as mean values +/- SD. **b** The total number of mutations in HPV- HNSCC is greater than in HPV+ HNSCC (3 ELF3 mutations were identified in HPV- HNSCC and 1 in HPV+ HNSCC). Differences in ELF3 mRNA expression between HPV- and HPV+ HNSCC are therefore not be explained by mutations in ELF3 (two-tailed unpaired t-test with Welch's correction, p=0.0005). **c** ELF3 mRNA expression as a function of ELF3 copy number (diploid, gain, shallow deletion, or amplification) shows that the majority (55 of 72) of HPV+ HNSCC tumors have normal, diploid copies of ELF3 despite increased ELF3 gene expression, indicating that increased expression is not explained by gene copy number. **d** HIDDEN cell markers and ELF3-induced targets MACC1 and GPR110 show increased mRNA expression in HPV+ HNSCC (two-tailed Mann Whitney test, p <0.0001). Source data are provided in the Source Data file.

	TCGA, Cervical SCC		TCGA, HNSCC	
	Log2 OR	q-Value	Log2 OR	q-Value
GPR110	>3	0.002	>3	<0.001
TMC4	2.9	0.003	2.5	<0.001
TMPRSS4	>3	0.004	>3	<0.001
TMC5	2.9	0.007	>3	<0.001
CLDN7	>3	0.007	>3	<0.001
GABRP	>3	0.01	>3	<0.001
LLGL2	2.3	0.018	2.3	<0.001
ERBB3	2.2	0.023	>3	<0.001
CLDN4	2.72	0.032	>3	<0.001
MACC1	2.3	0.039	>3	<0.001
MYO5B	2.6	0.039	>3	<0.001
RAB11FIP1	2.1	0.041	>3	<0.001
CEACAM6	2.2	0.046	>3	<0.001
MUC1	1.8	0.059	2.7	0.002
PDE4C	2.5	0.066	2.2	0.013
ERBB2	1.5	0.074	2.3	0.002
BCAS1	1.7	0.087	>3	<0.001

Supplementary Table 1. Core signature of HIDDEN cells refined by TCGA analysis. Genes found to co-occur with ELF3 at a statistically significant level) in TCGA Pancancer Atlas Cervical SCC and HNSCC cohorts using a mutual exclusivity test (one-sided Fisher's exact test $p<0.1$) evaluating the odds ratio (OR) of co-occurrence over baseline. Hits were sorted by q-value in the Cervical SCC dataset and yielded 17 genes. Including ELF3, this provided 18 co-occurring biomarkers of HIDDEN cells in subsequent studies. Source data is available in Data S5.

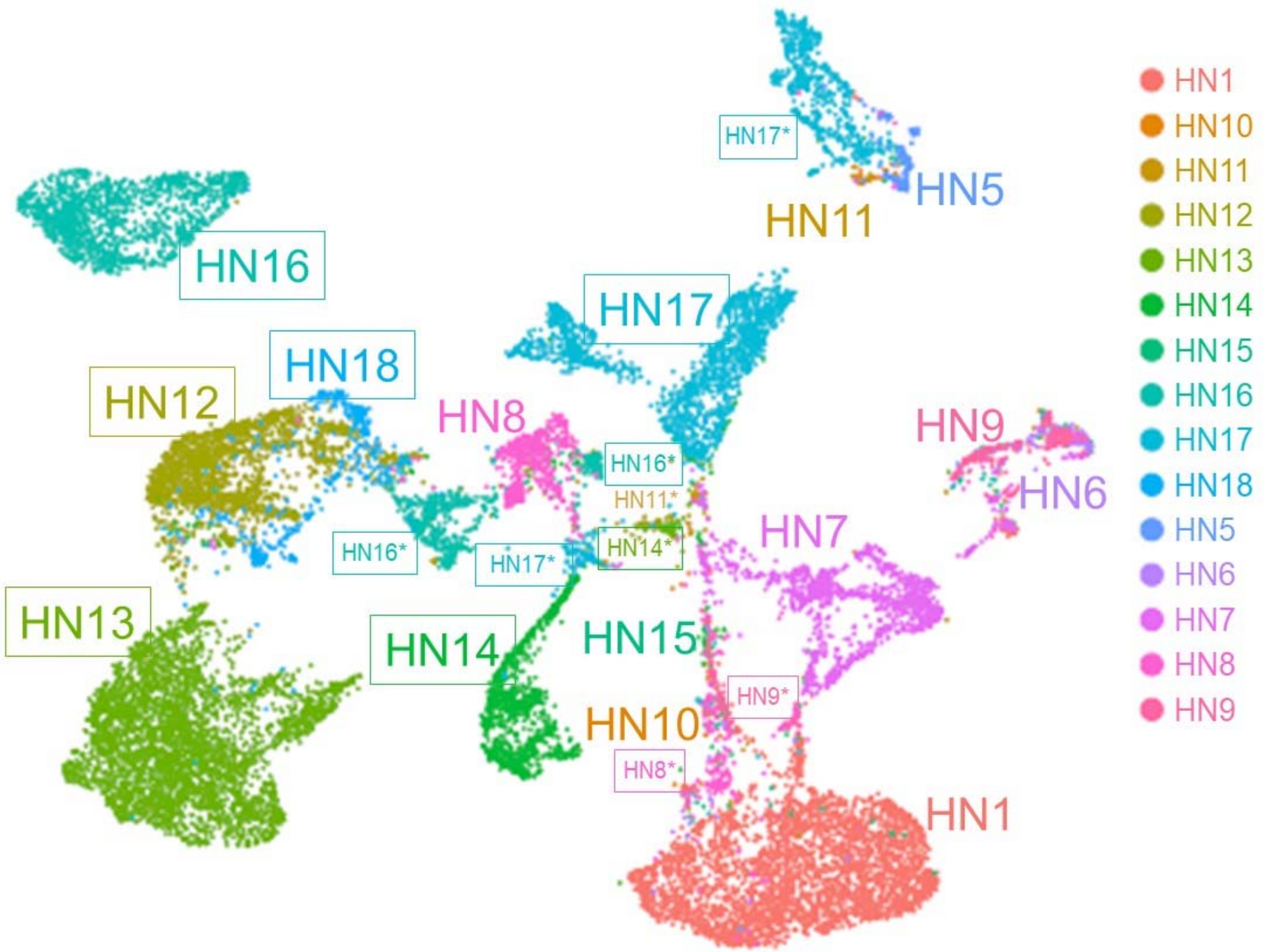

Supplementary Figure 5. In the absence of batch correction, HNSCC epithelial cells cluster primarily by tumor specimen. UMAP of clustered epithelial cells colored by HNSCC specimen demonstrates that patient variability results in little overlap between tumors (9 HPV- and 6 HPV+ tumors). HPV+ tumors are indicated by boxes around the labels. Smaller labels with an asterisk denote smaller sub-populations separated from the main cell grouping of specimens.

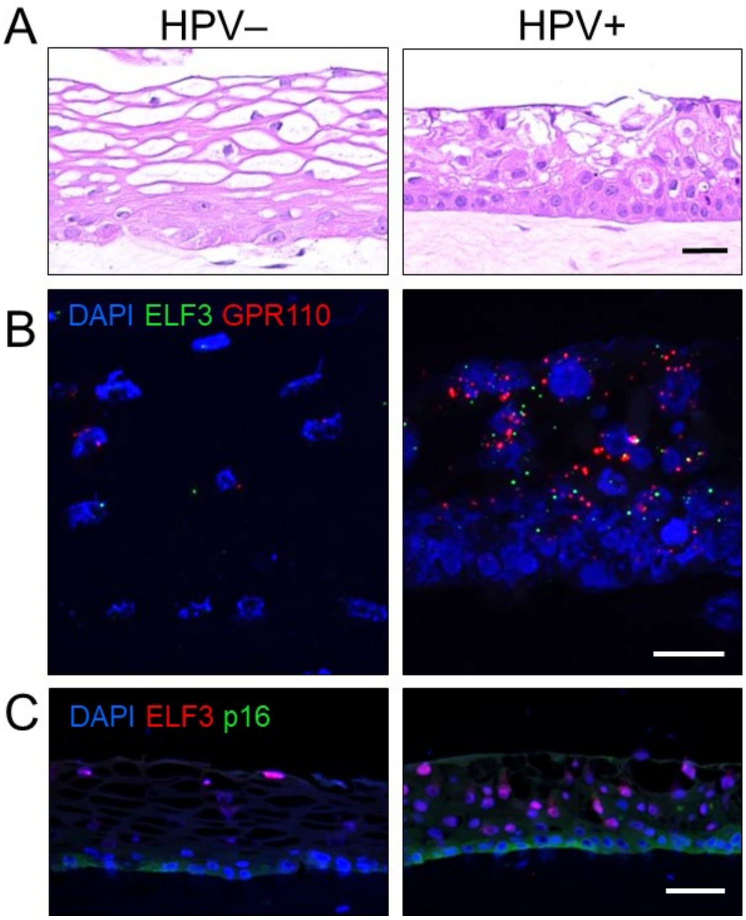

Supplementary Figure 6. A second set of tonsillar rafts derived from donor B further confirms HIDDEN cell upregulation. (a-c) Representative images from 10 frames across each raft set (n=1). **a** Representative H&E generated from tonsil-derived keratinocytes from a second patient (Donor B) that were either nucleofected with HPV16 or not nucleofected, independently from the patient used in Fig 9. Scale bar, 100µm. Similar to donor A, RNA-ISH for ELF3 and GPR110 (**b**, scale bar, 50µm) and IF for ELF3 (**c**, scale bar, 100µm) reveals significant upregulation of HIDDEN cells in the suprabasal compartment.

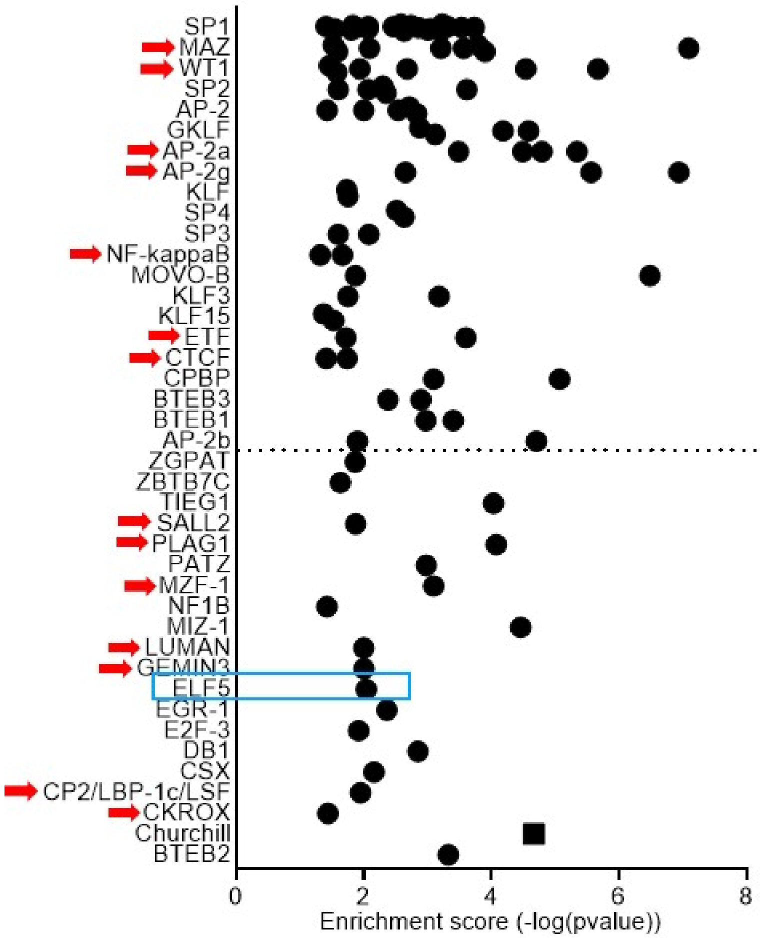

Supplementary Figure 7. Transfac analysis. Summary of Transfac analysis generated through the gProlifer web server, as detailed in Methods, which uses a tailor-made algorithm for statistical testing. Promoters of the HIDDEN cell specific transcriptome were scanned for enriched transcription factor motifs. Each dot represents one distinct motif identified as a potential regulator, with some transcription factors (e.g. those above the dotted line) harboring multiple motifs. Transcription factor motifs that were specifically identified within the ELF3 promoter are identified by red arrows. ELF5 is highlighted with a box.
