# Supplementary material for: Single cell transcriptomic analysis of HPV16-infected epithelium identifies a keratinocyte subpopulation implicated in cancer
Source: Nat Commun. 2023 Apr 8;14:1975. doi: 10.1038/s41467-023-37377-0 (PMC10082832; doi:10.1038/s41467-023-37377-0)
Supplement: Supplementary file 3 — Description of Additional Supplementary Files [file 41467_2023_37377_MOESM3_ESM.pdf]

File Name: Supplementary Data 1

Description: Method details related to antibodies, RNA-ISH probes, and RT-qPCR primers

File Name: Supplementary Data 2

Description: Gene lists for scRNAseq cluster, heatmap, and pseudotime analyses

File Name: Supplementary Data 3

Description: Gene lists of potential cluster-defining markers for C3 & C9, and RT-qPCR validation of selected genes

File Name: Supplementary Data 4

Description: GO term analyses for C9 ontologies and differential expression

File Name: Supplementary Data 5

Description: TCGA data analysis

File Name: Supplementary Data 6

Description: HOMER/RELI analyses
