# Supplementary material for: Single cell transcriptomic analysis of HPV16-infected epithelium identifies a keratinocyte subpopulation implicated in cancer
Source: Nat Commun. 2023 Apr 8;14:1975. doi: 10.1038/s41467-023-37377-0 (PMC10082832; doi:10.1038/s41467-023-37377-0)
Supplement: Supplementary file 10 — Reporting Summary [file 41467_2023_37377_MOESM10_ESM.pdf]

nature portfolio

Corresponding author(s): Mike Adam, Steven Potter, Susanne Wells

Last updated by author(s): 3/13/2023

Reporting Summary

Nature Portfolio wishes to improve the reproducibility of the work that we publish. This form provides structure for consistency and transparency in reporting. For further information on Nature Portfolio policies, see our Editorial Policies and the Editorial Policy Checklist.

Statistics

For all statistical analyses, confirm that the following items are present in the figure legend, table legend, main text, or Methods section.

n/a	Confirmed	
[ ]	[x]	The exact sample size (*n*) for each experimental group/condition, given as a discrete number and unit of measurement
[ ]	[x]	A statement on whether measurements were taken from distinct samples or whether the same sample was measured repeatedly
[ ]	[x]	The statistical test(s) used AND whether they are one- or two-sided<br>*Only common tests should be described solely by name; describe more complex techniques in the Methods section.*
[x]	[ ]	A description of all covariates tested
[ ]	[x]	A description of any assumptions or corrections, such as tests of normality and adjustment for multiple comparisons
[ ]	[x]	A full description of the statistical parameters including central tendency (e.g. means) or other basic estimates (e.g. regression coefficient) AND variation (e.g. standard deviation) or associated estimates of uncertainty (e.g. confidence intervals)
[ ]	[x]	For null hypothesis testing, the test statistic (e.g. *F*, *t*, *r*) with confidence intervals, effect sizes, degrees of freedom and *P* value noted<br>*Give P values as exact values whenever suitable.*
[x]	[ ]	For Bayesian analysis, information on the choice of priors and Markov chain Monte Carlo settings
[x]	[ ]	For hierarchical and complex designs, identification of the appropriate level for tests and full reporting of outcomes
[ ]	[x]	Estimates of effect sizes (e.g. Cohen's *d*, Pearson's *r*), indicating how they were calculated

Our web collection on statistics for biologists contains articles on many of the points above.

Software and code

Policy information about availability of computer code

Data collection	RT-qPCR was performed on an Applied Biosystems Step One Plus real-time PCR Stem with StepOne software v2.2. For single cell RNA sequencing, the 10x Genomics Chromium genomic sequencing platform CellRanger v3.0.2 was used.
Data analysis	Confocal imaging data was quantified in Imaris v9.9.1. All graphs and statistical analyses were generated in Graph Pad Prism v09. Single cell RNA sequencing fastq files were processed on the 10x genomics pipeline (CellRanger v3.0.2) on the Seurat R package.

For manuscripts utilizing custom algorithms or software that are central to the research but not yet described in published literature, software must be made available to editors and reviewers. We strongly encourage code deposition in a community repository (e.g. GitHub). See the Nature Portfolio guidelines for submitting code & software for further information.

Data

Policy information about availability of data

All manuscripts must include a data availability statement. This statement should provide the following information, where applicable:

- Accession codes, unique identifiers, or web links for publicly available datasets
- A description of any restrictions on data availability
- For clinical datasets or third party data, please ensure that the statement adheres to our policy

The RT-qPCR data generated in this study are provided in the Source Data file. The scRNAseq data generated in this study have been deposited in the GEO database under accession code GSE189670 [https://www.ncbi.nlm.nih.gov/geo/query/acc.cgi?acc=GSE189670]. The analyzed scRNAseq data are available in Supplementary Information files. The scRNAseq publicly available data used in this study are available on the Gene Expression Omnibus database: accession ID GSE164690. [https://

www.ncbi.nlm.nih.gov/geo/query/acc.cgi?acc=GSE164690].[38] TCGA data were accessed and are available through cBioPortal. [92,93]. The remaining data are provided with this paper within the Article, Supplementary Information or Source Data file.

Human research participants

Policy information about studies involving human research participants and Sex and Gender in Research.

Reporting on sex and gender	Patient biopsies used in all studies were de-identified prior to transfer to the research laboratory, and thus the distribution of sex/gender is unknown, with the exception of cervical tissues which by definition originate from only female patients.
Population characteristics	See above, these are also unknown
Recruitment	Patients were undergoing clinically indicated procedures that involved removal of tissues for treatment of disease (for example, excision of dysplastic or malignant tissues). Excess tissue was provided to support the studies.
Ethics oversight	University of Cincinnati, Cincinnati Children's Hospital Medical Center, or the University of Arizona, depending on the study, as detailed in Methods

Note that full information on the approval of the study protocol must also be provided in the manuscript.

Field-specific reporting

Please select the one below that is the best fit for your research. If you are not sure, read the appropriate sections before making your selection.

☒ Life sciences ☐ Behavioural & social sciences ☐ Ecological, evolutionary & environmental sciences

For a reference copy of the document with all sections, see nature.com/documents/nr-reporting-summary-flat.pdf

Life sciences study design

All studies must disclose on these points even when the disclosure is negative.

Sample size	No sample size calculation was performed. For RT-qPCR studies to determine expression prior to RNA-ISH validation, n=2 was determined to be sufficient as no statistics were performed. For RNA-ISH quantificiations, n=10 corresponded to approximately the entire distance of the raft, and n=3 rafts were performed to allow statistically significant comparisons. For experiments in patient biopsies (n=2, qualitative), these are initial translational studies limited by availability of tissues and provide proof of concept to support more in-depth future studies, rather than supporting any quantification.
Data exclusions	No data was excluded from this study
Replication	One set of matched HPV16- and HPV16+ rafts generated from isogenic NIKS lines were harvested on day 14 post plating, dissociated into single cells separately but in parallel, and subjected to single cell sequencing. N=2 sets of rafts were used in RT-qPCR experiments for confirmation of gene expression in samples in order to select genes for RNA-ISH validation. n=3 sets of rafts was used for RNA-ISH validations. n=2 patient biopsies per clinical category/condition was included in translational studies. All attempts at replication were successful.
Randomization	Samples were assigned to groups based on HPV16 gene expression i.e HPV16 protein/RNA or p16 status (clinical samples) in line with the study goals of comparing of HPV+ vs HPV- samples.
Blinding	Pathological review of patient biopsies (eg cervical tissue/CINs) was blinded. The remaining studies do not involve clinical research.

Reporting for specific materials, systems and methods

We require information from authors about some types of materials, experimental systems and methods used in many studies. Here, indicate whether each material, system or method listed is relevant to your study. If you are not sure if a list item applies to your research, read the appropriate section before selecting a response.

Materials & experimental systems

n/a	Involved in the study
	☒ Antibodies
	☒ Eukaryotic cell lines
☒	☐ Palaeontology and archaeology
☒	☐ Animals and other organisms
☒	☐ Clinical data
☒	☐ Dual use research of concern

Methods

n/a	Involved in the study
☒	☐ ChIP-seq
☒	☐ Flow cytometry
☒	☐ MRI-based neuroimaging

Antibodies

Antibodies used

p16 - Santa Cruz Mouse sc-56330; 1:150
EXPO5 - Abcam Rabbit ab129006; 1:200
COL17A - Abcam Rabbit ab184996; 1:200
SBSN - Millipore Sigma Rabbit HPA067734; 1:200
CARD18 - Millipore Sigma Rabbit HPA038582; 1:100
KLK5 - Millipore Sigma Rabbit HPA014343; 1:50
ELF3 - Millipore Sigma Rabbit HPA003479; 1:200 (IF) or 1:1000 (Western)
Rhodamine anti-actin - Biorad 12004163; 1:5000
K10 - abcam Mouse ab9025; 1:2000
LCN2 - R&D systems Rat MAB1757; 1:10
K17 - ProteinTech Rabbit 18502-1-AP; 1:200
All information on antibodies are provided in Supplementary Data S1.

Validation

Antibody information, including dilutions and validations, are detailed in Supplementary Data S1.

Eukaryotic cell lines

Policy information about cell lines and Sex and Gender in Research

Cell line source(s)

1) Spontaneously immortalized human keratinocyte cell line, NIKS. Provided by Paul Lambert, University of Wisconsin
2) HPV16 infected NIKS. Provided by Paul Lambert, University of Wisconsin
3) J23T3, commercially available. Provided by Paul Lambert, University of Wisconsin
4) Patient-derived cervical and tonsillar epithelial cell populations were directly cultured from biopsies and are not cell lines

Authentication

Southern blot analysis of episomal HPV16, PCR of HPV16 viral genes, DNAish of high risk HPV, RNAish of HPV16 genes. STR profiling was performed on NIKS and HPV16+ NIKS and compared to the original NIKS obtained from Dr. Lambert. J23T3 cells were not profiled since a variety of fibroblasts can be used for the support of keratinocyte growth.

Mycoplasma contamination

Cells were negative for mycoplasma by PCR and gel electrophoresis

Commonly misidentified lines
(See ICLAC register)

No cell lines used are categorized as commonly misidentified lines.
